# A Practical Treatise on Materia Medica and Therapeutics

**Published:** 1888-01

**Authors:** 


**A Practical Treatise on Materia Medica and Therapeutics.** By ROBERTS BARTHOLOW, M. A., M.D., LL.D., Professor Materia Medica and General Therapeutics Jefferson Medical College. Sixth edition; revised and enlarged. New York: D. Appleton & Co. 1887.

Bartholow's "Materia Medica" needs no praise. It is one of the necessities in every physician's library. The present edition contains a good deal of new matter. Most of the new remedies which have been proved to be of value, have been given a place, but he is careful to weigh the value of these new agents, of which he says: "Some possess the virtues assigned them—others seem hardly to justify their appearance in the *role* of remedy—and not a few, probably, are worthless for any purpose. The earliest announcements are not unfrequently given forth by medical *quidnuncs*, who are more enthusiastic than logical, more sensational than exact, and more concerned to advertise their own doings than to give expression to faithful observations." After a review of the results obtained by Bergeon's treatment of phthisis by gas enemata, he concludes: "There appears to be a growing conviction that those phthisical subjects who have experienced much benefit from the treatment, owed this result to the influences so strongly affecting the imagination of both practitioner and patient."

